# Viruses Infecting the European Catfish (*Silurus glanis*)

**DOI:** 10.3390/v13091865

**Published:** 2021-09-18

**Authors:** Mona Saleh, Boglárka Sellyei, Gyula Kovács, Csaba Székely

**Affiliations:** 1 Clinical Division of Fish Medicine, University of Veterinary Medicine, 1220 Vienna, Austria; 2 Fish Pathology and Parasitology Research Team, Veterinary Medical Research Institute, Hungária krt. 21., 1143 Budapest, Hungary; sellyei.boglarka@atk.hu (B.S.); szekely.csaba@vmri.hu (C.S.); 3 Research Institute for Fisheries and Aquaculture (HAKI), Hungarian University of Agriculture and Life Sciences, Anna-liget utca 35., 5540 Szarvas, Hungary; Kovacs.Gyula@uni-mate.hu

**Keywords:** iridoviruses, *Ranavirus*, *Circovirus*, *Herpesvirus*, *Rhabdovirus*, papillomaviruses

## Abstract

In aquaculture, disease management and pathogen control are key for a successful fish farming industry. In past years, European catfish farming has been flourishing. However, devastating fish pathogens including limiting fish viruses are considered a big threat to further expanding of the industry. Even though mainly the ranavirus (*Iridoviridea*) and circovirus (*Circoviridea*) infections are considered well- described in European catfish, more other agents including herpes-, rhabdo or papillomaviruses are also observed in the tissues of catfish with or without any symptoms. The etiological role of these viruses has been unclear until now. Hence, there is a requisite for more detailed information about the latter and the development of preventive and therapeutic approaches to complete them. In this review, we summarize recent knowledge about viruses that affect the European catfish and describe their origin, distribution, molecular characterisation, and phylogenetic classification. We also highlight the knowledge gaps, which need more in-depth investigations in the future.

## 1. Introduction

European catfish (also known as sheatfish or wels) *Silurus glanis*, is Europe’s largest freshwater fish species. The biggest individuals, in the past couple of decades, reached 2.75 m and 130 kg [1]; nevertheless in the past centuries, there were reports on catches over 200 kg and 3 m size fish [2]. The original habitat of wels ranged from Eastern Europe to the Ural Mountain; and from Sweden and Estonia in the North, to Greece, Turkey, Northern Iran, and Tajikistan in the South [3]. As other catfishes, e.g., Channel catfish, African catfish or pangasius, wels has no intermuscular bones and is an enjoyed fish. The majority of wels production comes from catches but its aquaculture is rapidly growing [4].

Wels is a top predator that grows fast in suitable conditions. In traditional carp farms, it was raised as useful accompanying fish when stocked less than 2 percent of fish in the pond. It reduces the number of white fish (the concurrent competitor of carp food), eliminates the diseased carp, and in this way decreases the chance of an outbreak of infectious fish diseases. In sparsely populated carp ponds, catfish are unlikely to infect each other. In the previous decades, a deadly infectious ectoparasite, *Ichthyophthirius multifiliis* (ich) caused significant difficulties in fingerling production. Formerly, malachite green, an organic compound was efficiently applied against ich, but its use is no longer permitted in aquaculture [5]. This is one of the reasons why with the spreading of intensive catfish farming methods today, fingerling production is increasingly taking place in closed systems where the appearance of ectoparasites is ruled out (https://cordis, 2019, accessed on 15 August 2021).

In intensive culture conditions, in a cage, tank, or heavily stocked earthen pond, the chance of mass infection grows significantly compared to that in extensive ponds. In closed systems such as recirculating aquaculture system (RAS), the major health problems for wels are not due to ectoparasites but infectious bacteria and viruses.

The knowledge about viral diseases or infections of catfish (*Silurus glanis*) is very limited in the scientific literature. Among the published cases, mainly the ranavirus (*Iridoviridea*) and circovirus (*Circoviridea*) infections are considered well- described, although these are generally based on only a few observations. Sporadically, other agents resembled herpes-, rhabdo or papillomaviruses are also observed in the tissues of catfish with or without any symptoms (haemorrhage, exophthalmos, and melanosis). The etiological role of any of these viruses has been unclear until now.

In particular, there is a need for better knowledge of the latter and the development of preventive and curative methods against them.

## 2. Iridovirus Infections

Viruses of the family *Iridoviridae* are cytoplasmic viruses containing a large dsDNA genome that have a host range restricted to insects and ectothermic vertebrates, fish, amphibians and reptiles [6].

Iridovirus virions are large, usually 120 (the naked)—200 nm (particles with external membrane) in diameter, but may reach up to 350 nm. The virions have icosahedral symmetry with hexagonal cross-section and complex structure [7]. From the outside inwards, the particles include an external membrane composed of lipid and glycoprotein that obtained released virions by the budding from the host cell membrane. This outer envelope is not released during cell lysis and is not essential for infectivity. The next inside layer is viral capsid constructed mainly (40%) from the highly conserved major capsid protein (MCP) with approximately 36 additional polypeptides, ranging from about 5 to 250 kDa. The outer surface of the capsid is covered by flexible fibrils or fibers of unknown functions with terminal knobs; and by additional structural transmembrane proteins that are integrated into the internal limiting lipid membrane surrounding the inner surface of the capsid shell [8].

Central to the inner core is the single, linear, double-stranded DNA genome that is packaged with associated proteins as a nucleoprotein filament. The genome is large, between 105 and 200 kilobase pairs (kbp), its G + C content approximately 49 to 54%. It is highly methylated and has circular permutation and terminal redundancy. This genome encodes approximately 100 viral proteins, depending on the genus, the majority of which have unknown functions and are unique to the virus family [9]. According to Eaton et al. [10], there are 26 (7 genes of them identified after genome re-analysis) core genes that are conserved across the family (Table 1 and Table 2); the diversity in the remainder reflects a broad range of hosts and environments.

**Figure 1:**
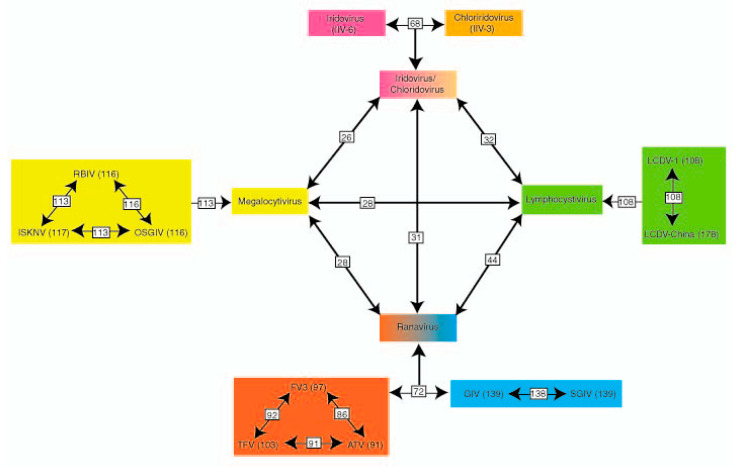
Phylogenetic relationships between iridovirus genera (shown in different colors) based on gene content (Eaton et al., 2007). Phylogenetic relationships between the five iridovirus genera based on gene content. The viral species were compared within a genus to identify the number of orthologous genes. Orthologous genes between viral genera were then specified. The numbers on each line identify the number of orthologous genes shared among viral species or genera comprising the 26 core genes. The *Iridovirus* and *Chloriridovirus* genera have a high level of gene conservation and a shared genera box (*Iridovirus*/*Chloriridovirus*) was used to compare orthologous genes between genera.

There are currently 2 subfamilies (*Alpha-, Betairidovirinea*), and 7 recognized genera within the family *Iridoviridae*. The family *Iridoviridae* has been divided based on morphological and biological distinctions. The *Iridoviridae* genera are serologically distinct and nucleotide sequence information indicates differences in gene content showing differences in amino acid identity greater than 50% in the MCP gene (Figure 1) [6].

Members of the genera *Iridovirus* and *Chloriridovirus* infect invertebrates, i.e., insects and crustaceans. *Ranavirus*, *Lymphocystivirus*, and *Megalocytivirus* genera are relevant to fish health. The member of *Lymphocystivirus* and *Megalocytivirus* infect fish species exclusively, but viruses in the genus *Ranavirus* have been associated with mortality events in amphibians, and reptiles, as well. Based on extended molecular studies, among the ranaviruses (RVs) are the grouper (GIV)-like RV and the Santee Cooper-like RV (taxonomy has been long controversial), and the amphibian-like ranavirus (ALRV) subspecies [10,11,12]. The ALRV comprises three groups: Epizootic hematopoietic necrosis virus (EHNV)-like, common midwife toad virus (CMTV)-like, and frog3 virus (FV3)-like ranaviruses [13]. The members of the ALRV subspecies rarely cause disease in fish, with the exception of pathogens of freshwater species such as the EHNV; the European sheatfish virus (ESV), and the European catfish virus (ECV), which spread is restricted to Australia and continental Europe, respectively.

The first case of RV infection was detected in sheat/catfish (*Silurus glanis*) in 1988 in Germany, where sheatfish fry of a commercial warm water recirculation aquaculture unit presented cumulative mortalities of up to 100% with unspecific clinical symptoms. The isolated (European) sheatfish (irido) virus—SFIR or SFV, then ESV) virus showed high virulence that resulted in the sudden death of fries by transmission via water bath within 8 days and co-habitation within 11 days in laboratory trials [14,15]. Sheatfish fry exposed to the water-borne virus exhibited anorexia, apathy, and ataxia, and they gathered at the heater. Generally, moribund fish moved slowly but occasionally they also showed sudden, rapid spiral movements. Gross signs of the infection were petechial haemorrhages in the skin and were occasionally present in eyes, barbels, and serosal surfaces. At necropsy, the liver and spleen of infected sheatfish were often swollen and pale, and there was no food in the digestive tract [16].

**Figure 2:**
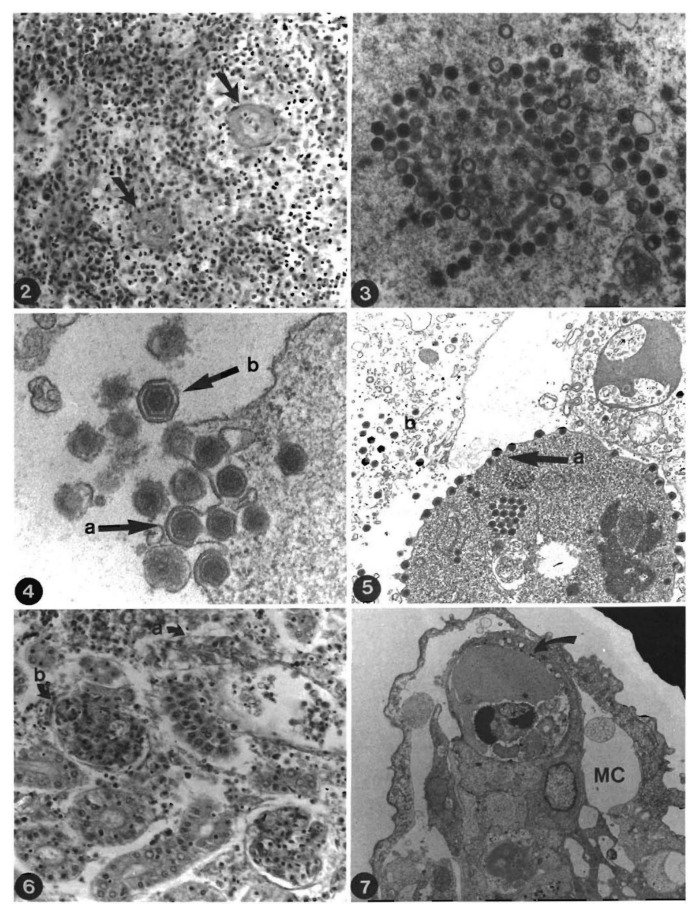
*Silurus glanis* pathological alterations. ② ([17]) Spleen. Degeneration of vascular walls (arrows) and parenchymal haematopoietic necrosis H and E. ×300. ③ ([17]) Spleen. Complete and incomplete virus particles within the cytoplasm. TEM, ×27,400. ④ ([17]) Spleen. Viral particles receiving envelope by budding from the plasma membrane. a Budding process. b released virion TEM, ×51,200. ⑤ ([17]) Kidney. a: Release of enveloped viruses from the cell; b lytic area with non-enveloped virus particles. TEM. ×14,700. ⑥ ([17]) Kidney. Renal excretory system. Degeneration of (a) renal tubules; (b) renal corpuscle H and E. ×300. ⑦ ([17]) Gills, secondary lamella. Virus particles (arrow) endothelium of marginal channel (MC) Oe-dema of respiratory epithelium with separation from basement membrane TEM, ×4900 (Ogawa et al., 1990; with permission; “©Inter-Research 1990”).

In the histopathological study, all parenchymatous organs showed pathological alterations [17] (Figure 2). The kidney and spleen are apparently the major target organs of the infection. ESV proved to be endotheliotropic, the multiplication of the virus in the endothelium results in a loosening of the capillary walls and leads to an increase in the permeability of blood vessels. In addition to effects on the endothelial cells, generalized destruction of the haematopoietic tissue of the kidney and spleen was observed. The result is oedema, haemorrhagic diathesis and peripheral circulatory failure. Moreover, the preliminary immunological study demonstrated a strong suppressive influence of ESV on pronephric macrophage and lymphocyte activity [18]. Persistence without signs of any disease and infectivity of this virus to adult sheatfish (cca. 1500 g) in a stress situation (presence of a high abundance of *Aeromonas hydrophila*) were also reported, with losses was up to 30% [19].

It is noteworthy that new ESV infections in seathfish (adult 2400–3100 g) was detected in 2014 in Poland [20]. In the meantime, a very similar ranavirus with almost identical clinical signs caused mass mortality among catfish stocks across Europe. The European catfish virus (CFIR, or CFV then ECV) was first isolated in France [21], then in Italy [22], then detected repeatedly in France [23] in some lakes where the black bullhead catfish ((*Ictalurus nebuluosus*) *Ameiurus melas*) was overpopulated. The two ranaviruses, ESV and ECV have been considered identical for a long time [10,24,25,26,27] but the differences in their sequence of neurofilament triplet H1-like (NF-H1) protein [28], and in their range of susceptible fishes [29,30] demolished this hypothesis.

The molecular characterisation and phylogenetic classification of ESV (and ECV) have been based on REA and sequencing of partial gene regions for about 30 years. Mavian et al. [31] reported the first whole genome of the European Sheatfish Virus. It belongs to the amphibian-like ranaviruses and is closely related to the epizootic hematopoietic necrosis virus (EHNV). The final genome sequence of ESV was slightly longer than that of the EHNV genome with a GC content of 54.23%. It has the highest degree of conservation with EHNV (88%) and *Ambystoma tigrinum* virus (ATV) (79%). A total of 136 putative open reading frames, including all of the iridovirus- [10] and ranavirus-specific genes as well as 12 out of 13 ALRV-specific genes [32], were identified.

Fehér et al. [33] sequenced and published the whole genomes of two Hungarian isolates that were highly similar (99.7–99.9% nucleotide identity) to ESV [31], however, these viruses were isolated from subadult and adult brown bullhead catfish (*Ameiurus nebulosus*) at separated water sources in Hungary [34].

## 3. Circovirus Infections

The family *Circoviridae* was established in the mid-1990 s when it was recognized that animal viruses with circular, single-stranded DNA (ssDNA) genomes were distinct from other eukaryotic ssDNA viruses classified at the time. In fact, until 2010, only a diverse group of avian species were known to be affected by circoviruses, except for pigs. Later, studies employing viral metagenomic-based strategies and degenerate PCR for circoviruses in unconventional hosts have since identified the presence of circovirus genomes in freshwater fish and various mammals (including bats, chimpanzees, dogs, humans, and minks), and invertebrates [35]. It should be noted that whole-genome sequencing processed recently have shown that circovirus-derived sequences such as endogenous viral elements (CVe) can be detected in several eukaryotic genomes as well [36].

The family historically is divided into two genera, *Circovirus*, which originally contains the avian and porcine pathogens; and *Gyrovirus*, which includes a single species (Chicken anemia virus). Later, with the increasing number of the recognition and genomic characterisation of more genomes from different host species, a new taxonomic unit namely the *Cyclovirus* genus has been introduced within *Circoviridea*. Even though the genomes of viruses in the three genera exhibit several common features, the significant divergence detected in the chicken anemia virus such as negative-sense genome organisation, led to the transfer of *Gyrovirus* into the family *Anelloviridae*.

Circoviruses including members of both *Circovirus* and *Cyclovirus* genus are small (15–25 nm), non-enveloped viruses with an icosahedral capsid containing a short single-stranded, covalently closed circular DNA genome of 1.8–2.1 kilobases. All circovirus genomes have an ambisense organisation containing open reading frames (ORFs) arranged on different strands of a dsDNA replicative form. The two major ORFs encoding the replication-associated protein (Rep) on the sense strand, and capsid protein (Cap) on the complementary strand of the replicative form. The Rep, which has sequence motifs characteristic of proteins involved in rolling circle replication, is the most conserved circovirus protein. On the other hand, the Cp is significantly divergent and is only characterised by an N-terminal region rich in basic amino acids that may provide DNA binding activity. These genomes are also characterised by the presence of a conserved nonanucleotide motif. This stretch is an essential part of the stem-loop structure of the circoviral genome marking the origin of replication in the 5′ intergenic region between rep and cap. Notable that the orientation of major ORFs relative to the nonanucleotide motif differs between genomes representing the two genera. In addition, in several circovirus species, other ORF with known or un-known functions have been reported [37].

Even though circovirus-related sequences of the *Circovirus* genus have been detected in several members of different orders of bony fish (*Anguilli*-, *Characi*-, *Clupei*-, *Cyprini*-, *Cyprinodonti*-, *Perci*-, and *Salmoniformes*) [36,38,39], the whole virus genomes could be derivated from barbel (*Barbus barbus*), European catfish (*Silurus glanis*) and European eel (*Anguilla anguilla*) up to now [40,41,42,43].

The catfish circovirus (CfCV) was the second fish circovirus that was discovered in fish [41]. During the spawning season, between May and July, of 2011, several maturated European catfish were found dead in Lake Balaton of Hungary in higher numbers than in previous years. The bodyweight of dead and moribund catfish were 6–50 kg. At the autopsy, dermal lesions and vascular dilations in the skin, and inflammation of the gastrointestinal tract were observed. The histopathological examinations revealed nuclear fragmentation in the hematopoietic cells raising the suspicion of some kind of undetected toxicosis. However, the routine bacteriological, parasitological, virological examinations, and toxicological tests did not give a real explanation for the mass mortality of fish. Using different tissue samples (liver, spleen, gills, kidneys, and gonads) of 6 moribund catfish, two full genome nucleotide sequences (CfCV-H5 and CfCV-H6, GenBank no.: JQ011377 and JQ011378) were generated from 2 different fish proved circovirus positive in the preliminary assays with nested PCR [44] and sequencing (with HT-F: 5′-CAGACCATGCTTCCGGTACT-3′, HT-R: 5′-GGGCTTCCTCGAAGGTTATC -3′ primer pair). The size of both genomes was 1966 nucleotides (nt) organised in a single-stranded DNA (ssDNA), forming a covalently closed circular molecule.

The nucleotide sequences of the two viruses were 99.4% identical, showing a low level of divergence. Two major open reading frames (ORFs) of opposite orientations, separated by short intergenic sequences (282 bases at the 5′ end and 60 bases at the 3′ end) were predicted. ORF1 is located between 251 and 1193 nt and has a potential coding capacity of 314 amino acids (aa), whereas ORF2 spans from 1934 to 1253 nt encoding 217 aa. In addition, further two smaller ORFs with unknown functions, ORF3 (158 aa, 472 bases, from nt 473 to 1) and ORF4 (136 aa, 408 bases, from nt 27 to 435) were identified in the genome. The genomes carry a conserved nonanucleotide 5′-TAGTATTAC-3′ sequence as a part of the stem-loop structure at the intergenic region between ORF1 and ORF2, characteristic for viruses of the *Circovirus* genus. However, the length of the double-stranded stem is only 9 bp for CfCV, shorter than the same region of most circoviruses, but similar to reported cyclovirus stems [45]. Analysis of CfCV ORF1 derived amino acid sequences indicated homology with other circovirus Rep proteins. The amino-terminal part of the potential capsid (Cap) protein of CfCV (coded by ORF2) carries a 31 aa long arginine rich stretch (RRRTFRRPIRRRMHRRTRGRRMIRRRSRRSR) starting at residue 4, homologous to the circovirus Cap nuclear localization signal. Based on comparisons of the derived amino acid sequences of both ORF1 and ORF2 with adequate homologous proteins in Blast, the closest relative of CfCV is the NG13 virus detected in human stool samples of children with acute flaccid paralysis [45], with 55% and 35% similarity, respectively. The molecular data indicate that the virion structure of CfCV is characteristic of circoviruses in general [46], which is constructed by single repetitive Cap proteins whose beta-sheet core configuration results in the icosahedral appearance.

Even though circoviruses were originally considered to be host-specific, detection of porcine circovirus (PCV) in alternative hosts [47,48,49] and European eel CV (EeCV) in the sichel [43] rather indicates that their host spectrums may be actually wider than it has been estimated before. The transmission of PVC and some avian viruses are known, where the fecal-oral route is common, but vertical transmission is also observed.

The ability of fish circoviruses to induce disease is not yet known. However, circoviruses mainly replicate in dividing cells, therefore young animals are more likely to develop the disease. It may have been the reason for the mass mortality of the barbel fry associated with BarCV [40]. Dividing cells for the infection are always available in the activated immune system, which is the reason why these viruses are considered to be strongly immunosuppressive [50]. It is also known that circoviruses are mostly present in animals in the form of subclinical infections. So the appearance of circovirus-associated clinical signs is not unusual when there is a deterioration of the physical condition. Such was the case of European catfish where increased mortality was reported during the exhaustive spawning season of 2011 [41]. More than 2000 moribund and dead large-sized sexually matured catfish were found in Hungary’s Lake Balaton. No clear link was established between the CfCV infections and mortality, but no other causative agent could be detected in these fish.

## 4. Herpesvirus Infections

Herpesviruses are host-specific pathogens, that are widespread among mammals, birds, and fish as well. Genetic analysis has shown three distinct (sub)families within the order *Herpesvirales*. The member of the *Herpesviridae* infects reptiles, birds, and mammals; the *Malacoherpesviridae* infects mollusks; and of the *Alloherpesviridae* infects fish and amphibians [51,52,53].

There is almost no sequence similarity between the families. In fact, in large-scale comparisons between the deduced amino acid sequences of the genes of the DNA viruses, the *Herpesvirales* did not form a monophyletic group [54]. The only gene with distinct homology is the ATPase subunit of the terminase protein, which is involved in packaging genome into the capsid during virion assembly [52,55].

In *Alloherpesviridae*, there are 12 genes that are consistently conserved [56]. Seven of these genes encode proteins are involved in basic structure or have essential functions in replication such as capsid morphogenesis (capsid triplex protein 2, capsid protease and scaffolding protein, and the major capsid protein), DNA replication (DNA helicase, DNA polymerase, and primase) and DNA packaging (ATPase subunit of the terminase). The other five conserved genes encode proteins with unknown functions.

The phylogenetic study based on the sequence of a portion of the DNA polymerase gene and ATPase subunit of the terminase gene shows that there are two monophyletic clades within this family, the Clade 1 with *Anguillid* (AngHV1)-, and *Cyprinid* herpesviruses (CyHV1, -2, -3); and the Clade 2 with *Ictalurid* (IcHV1, -2)-, *Acipenserid* (AciHV1, -2)-, *Salmonid* (SalHV1, -2, -3)- and *Ranid* (RaHV1, -2) herpesviruses [53]. The latter, Clade 2 could be subdivided into 2 further groups, one containing the frog herpesviruses and the other containing the fish herpesviruses of this clade [57]. The established genera and associated species are *Batrachovirus* (RaHV1, -2); *Cyprinivirus* (CyHV1, -2, -3), and AngHV1; *Ictalurivirus* (IcHV1, -2 and AciHV1, -2); and *Salmonivirus* (SalHV1, -2, -3) in *Alloherpesviridae* [58].

Despite the lack of sequence homology between the families, the basic structure and biological characteristics are identical.

Structurally, a herpesvirus virion contains a linear, double-stranded DNA genome (130–300 kb, that coding 70–160 genes) packaged densely within an icosahedral nucleocapsid (capsid diameter ~100 nm) that is surrounded by a proteinaceous tegument layer and finally a host-derived envelope [59]. The nucleocapsid is composed 162 capsomeres (150 hexons, 12 pentons) and each of them has a chimney-like protrusion with an axial channel in the middle [60,61]. The replication scheme of these viruses is also conserved [62].

The main biological characteristics shared by these viruses are a high level of host specificity, long-term latency, and epitheliotropism [63]. Thus, all known alloherpesviruses cause disease in only one species of fish or in closely related members of the same genus. In non-native hosts, they provoke abortive or only mild infection, but occasionally they could cause fatal disease. Their host specificity and recalcitration to propagation in cell culture due to lack of suitable cell line from the affected species explains the high number of fish herpesviruses that have not been characterised yet.

The other common characteristic of these viruses is that they establish a latent state in the host after initial replicative infection. During this latent state, the viral genomic DNA is detectable but infectious virus particles are not, and very few viral genes are expressed in the survivors of a productive primary infection. The regulation of latency and productive replication may be intricately correlated to the physiology of the host.

The clinical signs and outcome of disease vary substantially during primary and recurrent infections and are probably controlled by the dose of the virus and the immune status of the fish. In the primary infection, viruses often undergo productive replication in a variety of cell types of the natural host. This may result in either viremia and acute disease with high morbidity and mortality, or cause mild illness and virus dissemination within a population creating a mass of carrier survivors.

The most common manifestation of disease associated with alloherpesviruses is dermal or epidermal cell proliferation and lesion of the skin and/or gills, haemorrhages, occasionally ascites, and kidney and liver necrosis. These proliferative lesions are often unsightly but are rarely fatal.

Histopathologically, cell hypertrophy and kariomegaly or karyorrhexis by large eosinophilic to amphophilic intranuclear inclusions that marginate the chromatin often can be observed in the affected areas [64].

Pox-like epidermal changes (such as epidermal hyperplasia, papillomas) associated with mild or severe diseases caused by (allo)herpesviruses in at least 17 fish species have been published in the last 60 years. However, their host and tissue specificity make them recalcitrant to cell culture. Thus, there are many more disease-causing herpesviruses that have yet to be characterised. Herpesviruses-like infecting agents have been detected by electron microscopy but not cultured and not yet confirmed by molecular methods from 13 other fish species [63].

Pathological changes similar to carp pox in the sheatfish (*Silurus glanis*) were first reported by Lucky [65]. He gave the exact histopathology of the lesions, but the viral origin was only supposed. Clinical signs of the infection in this species had been known in Hungary as well for a long time, but no mortality due to the disease was observed until 1980. Then, heavy mortality associated with serious skin lesions was observed in a sheatfish population kept in net-cages for two years. The disease appeared during the winter of the second year, with the following gross pathology: a greyish, slightly elastic coat that covered the skin surface in regions or formed fluent sheath mostly on the head. The affected animals showed a generally slimy appearance. In the moribund fish, characteristic lesions at several spots of the skin and liver dystrophy, but no other changes in the organs were found. Pathological changes could be seen in two-thirds of the population and the mortality was about 50% during the winter season.

At the histopathological study of the tissues of affected fish, the light microscopy revealed distinct thickening in the dermis (stratum spongiosum, which together with the stratum basale forms the formed Malpighian layer). In some epidermal cells, enlarged nucleus contained central Cowdry-A type acidophilic (eosinophilic or basophilic) inclusion body was observed. These nuclei showed an irregular shape and were sometimes disintegrate. Acidophilic debris could also be seen in the pale cytoplasm. The proliferated epidermis contained almost no mucus cells at all. The degenerated upper cell layers seemed to be gradually detached from the deeper layers. Even though, based on unpublished observations, sometimes the virus infection stimulates the development of tumors that could reach deeper layers of the skin.

On electron micrographs, the chromatin density was higher around the membrane of the irregular nuclei. Virus particles of 85–90 nm in diameter were scattered in the nucleoplasm. The cytoplasm of these cells contained enveloped virus with a diameter of 145–160 nm. While autophagic vacuoles were seen in the cytoplasm of earlier degenerated epithelial cells.

The molecular confirmation of the role of any (allo)herpesvirus in the disease is missing as attempts to propagate the causative virus on cell lines have not been successful until now.

Ultrastructural changes and the virus detected in the above-mention case resembled that had been found in carp herpes infection. Apart from that, the degeneration of the epithelial cells in sheatfish was faster and more profound. This was also demonstrated by the high amount of cell debris found in the cytoplasm of the affected cells. This may be due to the fact that the desquamatation of the upper epidermal layers of the sheatfish’s skin is easier, and perhaps even the different cell structure makes it more prone to herpesviral infection [66].

## 5. Rhabdovirus Infections

The virus order *Mononegavirales* was established in 1991 to accommodate related viruses with non-segmented, linear, single-stranded, negative-sense RNA genomes. Today, the order includes together with *Rhabdoviridae*, 8 families [67].

The members of the family *Rhabdoviridae* bear typical bullet-shaped or bacilliform morphology in the range of 100–460 nm in length and 45–100 nm in diameter. Virions have helical symmetry and show cross-striations (spacing 4.5–5 nm) in negatively-stained thin sections. The ultrastructure of the inner nucleocapsid consists of a ribonucleoprotein (RNP) complex comprising the genomic RNA and tightly bound nucleoprotein (N) together with an RNA-directed RNA polymerase (L) and polymerase-associated phosphoprotein (P). Externally, it is surrounded by a lipid envelope containing glycoprotein molecules (G) that interact with the coiled RNP complex via the matrix protein (M). Notable, non-enveloped filamentous virions have also been reported [68].

Rhabdoviruses usually have unsegmented -sometimes bi-segmented- molecule(s) of linear (rarely hairpin), negative-sense (-) single-stranded RNA genomes (approximately 10–16 kb) with partially complementary non-coding termini. They generally encode the five major structural proteins (in the order 3′-N-P-M-G-L-5′) but may also encode additional (accessory) proteins either in additional genes or as alternative ORFs within the structural protein genes [69]. Genomes of viruses assigned to different genera may vary greatly in length and organisation.

Except for plant-infected nucleorhabdoviruses, replication of rhabdoviruses generally is happening in the cytoplasm. The process started with clathrin-mediated or receptor-mediated endocytosis with the contribution of G protein formed a spike and finished with the release (budding) through either the plasma membrane or internal membranes [70].

Rhabdoviruses are a large and ecologically diverse group of viruses, which infect terrestrial and aquatic vertebrates, invertebrates, and plants. Within the *Rhabdoviridae* 30 genera (and 191 species) have been established to date. Viruses assigned to each of the 30 genera form a monophyletic clade based on phylogenetic analysis of L sequences. They usually have similar genome architecture, including the number and locations of accessory genes, and have similarities in host range, modes of transmission, and/or sites of replication in the cell.

Rhabdoviruses of aquatic hosts include important fish pathogens in three genera: *Novirhabdovirus*, *Perhabdovirus*, and *Sprivivirus* [71]. These genera contain only viruses of freshwater and marine fishes. Due to the poikilothermic (cold-blooded) nature of their host, the replication temperature range for them is relatively low (15–25 °C). They are transmitted horizontally as waterborne viruses or vectorborne, and presumably vertically by egg-associated routes from parent to offspring. In each genus, the virus species are distinguished serologically based on the lack of cross neutralisation and molecularly—sequence divergence of partial G (or L—Perhabdovirus) gene sequences.

**Figure 3:**
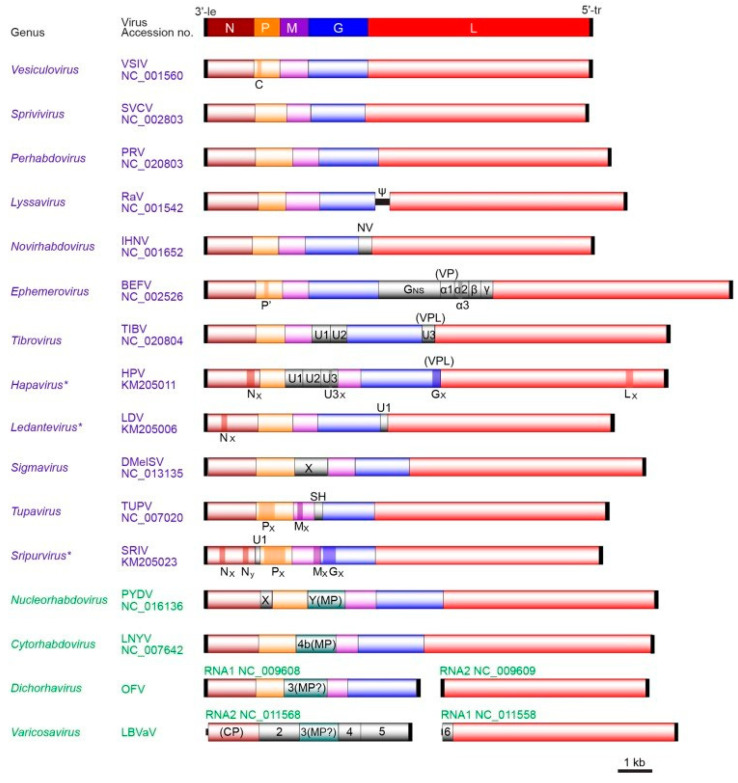
Comparative genome organisation of representative members from 16 genera of the family Rhabdoviridae. The five canonical structural protein genes (N, P, M, G and L) are presented in different colors. Other genes including movement protein (MP), viroporin (VP) or viroporin-like protein(VPL) and unknown function protein genes are seen in grey (Dietzgen et al., 2017 with permission).

The *Novirhabdovirus* genus includes 4 species: *Hirame novirhabdovirus*, *Piscine novirhabdovirus*, *Salmonid novirhabdovirus*, *Snakehead novirhabdovirus*. *Novirhabdovirus* genomes differ from those of the viruses in other fish rhabdovirus genera (3′-N-P-M-G-L-5′) in having an additional gene encoding a non-virion (NV) protein between the G and L genes [71,72,73]. (Figure 3). The NV protein is expressed at low levels in infected cells [74], where it localizes to the nucleus, interferes with the host interferon response [75], and triggers apoptosis [76].

The *Perhabdovirus*, and *Sprivivirus* genera currently have 3 (*Anguillid perhabdovirus*, *Perch perhabdovirus*, *Sea trout perhabdovirus*) and 2 (*Carp sprivivirus* (SVCV), *Pike fry sprivivirus* (PFRV) viral species, respectively). Virus genomes of both genera are relatively simple, containing only the five structural protein genes and short intergenic regions.

The Spriviviruses induce acute, contagious, systemic infections with generalized viraemia and haemorrhages in viscera and muscles. Even though the natural host of SVCV infections are predominantly cyprinid fish and the PFRV was isolated originally from pike, they occur in several different fish species, such as sheatfish.

Fijian et al. [77] published the first report on sheatfish fry mortality associated with rhabdovirus. The initial case of the red disease was followed by at least 6 outbreaks on 4 different fish farms between 1983 and 1985 in Hungary revealing the histopathology of this disease [78].

In all cases, the clinical signs were started among larvae, after uptaking the first feed on the 3th–4th days post-hatchery in the May-July period of the year. During the next 5–6 days, the mortality increased up to 100% in the stocks rearing in recirculation systems at 20–24 °C water temperature. The outbreaks have never affected fingerlings older than 6–8 weeks.

Sick fish were dark, and they had a slight to moderate pronounced hydropic syndrome. Mild haemorrhages in fins, gills, and edematous changes throughout the body, especially on the head were reported. At the autopsy of moribund fish, a large quantity of transparent serous blood-tinged ascitic fluid was found in the peritoneal cavities. Spleens were enlarged and bordered by haemorrhages. Livers were pale, and sometimes catarrhal enteritis was observed. The musculatures were not affected. The histopathological examination showed extensive haemorrhages under the capsule of the kidney. Nephrosis could be observed in the areas of the pro- and mesonephros. Different degrees of degeneration have been detected in convoluted renal tubular epithelial cells associated with rupture of the nuclear membrane, and foamed cytoplasm that occasionally was spotted with eosinophilic granulomas or cell debris. In some spots, this eosinophil cell debris appeared in the lumen of the renal tubuli, as well. In the interstitium of the kidney, increased number of red blood cells and focal debris accumulations presumably from necrotic lymphoid cells were observed.

In the hepatic parenchyma, rows of hepatocytes were disorganised. In the hepatic cells, the nucleolus became characteristic, its membrane was ruptured and its cytoplasm appeared to be vacuolated. In some sections, syncitia were formed by degenerated, contracted hepatocytes. The syncitia contained several nuclei and were characterised by a granular eosinophilic cytoplasm. Among mesenchymal cells, clues of an inflammatory reaction were observed. Macrophage centers containing ceroid pigments were enlarged and lymphocytes infiltrated the dilated portal tract. In sinusoids, Kupffer cells were swollen or partially degenerated.

In the central nervous system, the endothelium of blood capillaries was damaged, high amount of red blood cells were detected in the enlarged perivascular spaces. The lumen of the cerebral ventricles enlarged. Endothelial proliferation was recorded in the stomach and intestine, and dilatation was observed in the capillaries of the submucosa [78].

After a definite judgment on the role of the viral agent in the outbreaks, the isolation of the virus was successful using different cell lines such as epithelioma papulosum cyprini (EPC), fathead minnow (FHM), brown bullhead (BB). Morphology by electron microscopy and other characteristic properties of sheatfish isolate are typical for a rhabdovirus. Results of serological tests indicate a close relationship of this virus with SVCV [77].

Within the Sprivivirus genus, SVCV occurs as a single recognized serotype. On the other hand, SVCV antibodies cross-react to various degrees with viruses assigned to the species Pike sprivivirus. The immunochemical and biological examinations on 22 rhabdoviruses isolated from different fish species including four Hungarian isolates showed that viruses with serological similarity (in the absence of molecular studies) to both SVCV and FPRV species could be isolated from sheatfish [79]. Susceptibility of wels to SVCV infection was declared by several studies [80,81].

## 6. Papillomaviruses Infection

Papillomaviruses (PVs) are ecologically ubiquitous and ancient viruses infecting all studied vertebrates [82], from fishes to mammals. They form a large, diverse group of DNA viruses that are highly host-species-specific, although the distantly related virus types can infect the same host species as a result of co-evolution followed by virus-host-associated speciation [83].

Papillomaviruses are considered a part of the healthy skin microbiota [84]. They are epitheliotropic viruses that can induce infections with ample manifestation forms in the stratified squamous epithelia of skin and mucosal membrane [85]. The majority of them are non-pathogenic and cause subclinical infections in infected individuals or benign, often self-limiting papillomatous lesions, proliferative epithelial thickening with exophytic focus. A limited number of papillomavirus strains are oncogenic and are associated with the development of malignant neoplasms [86].

Various clinical symptoms may be due to the predisposition of these viruses to latent infection [87] when the viral DNA is persistent in the tissues at low copy numbers for long periods of time and new virus particles are not produced and released [88,89]. The life cycle of PVs is dependent on and intimately coordinated with cell replication and differentiation [90,91]. The infection starts when the virus gains access to a basal cell, probably due to microtrauma [90].

The virus is transported to the nucleus, and the production of early proteins results in (initial) limited amplification of viral DNA [91,92]. Later on, the infection is maintained by the proliferation of basal cells. However, terminal differentiation and keratinization of an infected cell are required for a productive infection [93]. The cell differentiation triggers the production of additional early proteins, which prevent the suprabasal cell from leaving the cell cycle (which would result in nuclear degeneration) and instead causes the cell to re-enter the S-phase of the cell cycle [91]. Eventually, these changes generate differentiation-dependent genome amplification and production of progeny virions [94,95,96]. As the infected cell reaches the upper epithelium, the PV late genes are expressed and viral assembly occurs close to the cell surface. PVs do not cause cell lysis, and viral particles are only released after the epithelial cell has been sloughed from the epithelial surface and degraded [93].

PVs are small, icosahedral, non-enveloped viruses that have circular double-stranded DNA genomes ranged between 5.7–8.6 kilobase [97]. The genomes are divided into three functional regions. The early (E) region encodes viral proteins—including the potential oncoproteins for some PV types [98,99]—involved in transcription, replication and manipulation of the cellular milieu. The late (L) region encodes the capsid proteins L1, and L2. The upstream regulatory or long codon region (URR or LCR) is located between the L1 and ORFs of early proteins and contains the viral origin of replication (ori) as well as binding sites for viral and cellular transcription factors [100]. A typical papillomavirus encodes six to nine proteins (L1–2, E1–8). However, the ancestral ones (piscine PVs) may have only contained a core set of four proteins (E1—DNA helicase, E2—master regulator, L1, and L2—capsid proteins). Phylogenetic classification of PVs is based originally on the global multiple sequences or pairwise alignments of the L1 genes, as one of a highly conserved ORF among the different PV types [101,102,103]. However, papillomavirus genera are primarily delineated by visual inspection of phylogenetic trees derived from concatenated E1, E2, L1, and L2 nucleotide sequences, in practice.

Papillomaviruses (PVs) are belonging to *Papillomaviridae* family [104]. The family includes two subfamilies, *Firstpapillomavirinae*, which consists of more than 50 genera and approximately 130 species, and *Secondpapillomavirinae*, with a single genus and species. To date, the single accepted piscine PV is the Alefpapillomavirus1 [105] from gilt-head (sea) bream (*Sparus aurata*) as an exclusive representative of the genus *Alefpapillomavirus*, that the only member of the subfamilies *Secondpapillomavirinae* [106]. However, Tisza et al. [107] have recently added 4 additional PV genomes isolated from fish (Haddock, Rainbow trout, and Red snapper) to the publicly accessible GenBank database as a partial result of metagenomic surveys.

Epidermal papillomas are commonly observed in many fish [108]. Skin diseases manifested in papillomas are also long known among sheatfish populations. Different viral aetiology (mainly herpesviruses) of these disorders has been supported [65,66] but their background is not always clear [109,110].

Recently, novel papillomavirus (PV) has been molecularly described and characterised in Hungary from farmed sheatfish showing multiple, papilloma-like, epidermal hyperplasia on the skin [111]. The complete genome of *Silurus glanis* papillomavirus 1 (SgPV1) is 5612-bp and it contains, similar to the previously described fish PVs only the minimal PV backbone genes (E1-E2-L2-L1) without any of the oncogenes (E5, E6, and E7).

The pairwise alignment of the L1 gene showed less than 60% identity with that of any other piscine PVs and the phylogenetic tree reconstruction illustrated separation of the new PV. Thus, the establishment of a novel genus (*Nunpapillomavirus)* within the subfamily *Secondpapillomavirinae* has been proposed for the species SgPV1 named *Nunpapillomavirus siluri*.

## 7. Concluding Remarks

The increasing global need for fish along with the limited availability of wild-capture to encounter this need has shown that fish farming is the only way to meet our demand worldwide. European catfish and a vast variety of aquatic animals are farmed in high density in freshwater, brackish, and marine systems, where they are exposed to various environmental and on-farm stressors, which compromise their ability to combat infection, and farming practices and enable the fast spread of disease. Viral pathogens of European catfish, whether they have been established for decades or if they are recently emerging as disease threats, are predominantly challenging since there are few if any, successful treatments, and the development of effective viral vaccines for delivery in aquatic systems remains elusive.

Molecular diagnostic tools of the European catfish viruses are developing, as the relevant molecular data about the ESV (European sheatfish viruses) as well as about (allo)herpesvirus and rhabdovirus in sheatfish are continually explored. Catfish viruses are routinely identified and/or characterised by electron-microscopy and traditional viral lab technics such as chloroform sensitivity, IUDR-iododeoxyuridine assay, and virus neutralization. Hence, to expand our ability to early detect more unidentified viruses in catfish more molecular investigations and characterisation are required. This can help us to timely detect devastating virus outbreaks enabling efficient disease management and control infections caused by viruses in aquaculture.

## Figures and Tables

**Table 1:** Iridovirus core genes.

	Gene Name ^a^	FV3	TFV	ATV	SGIV	GIV	LCDV-1	LCDV-C	ISKNV	RBIV	OSGIV	IIV-6	MIV
1.	Putative replication factor and/or DNA binding-packing	1R	105R	91R	116R	79R	162L	181R	61L	57L	60L	282R	79L
2.	DNA-dep RNA pol-II Largest subunit	8R	8R	6R	104L	71L	16L	191R	28L	29L	31L	176R, 343L	90L
3.	Putative NTPase I	9L	9L	7L	60R	36R	132L	075L	63L	59L	63L	22L	87L
4.	ATPase-like protein	15R	16R	83R	134L	90L	54R	114L	122R	116R	119R	75L	88R
5.	Helicase family	21L	21L	78R	54R	32R	6L	7L	56L	54L	57L	67R	4R
6.	D5 family NTPase involved in DNA replication	22R	22R	77L	52L	31L	128L	80L	109L	101L	106L	184R	121R
7.	Putative tyrosin kinase/lipopolysaccharide modifying enzyme	27R	29R	58R	**78L + 81L** ^b^	52L	195R	173R	61L,114L	57L, **106L** ^b^	60L, 111L	179R, 439L	35R
8.	NIF-NLI interacting factor	37R	40R	64R	61R	37R	82L	148L	5L	6L	6L	355R	104R
9.	Unknown	41R	45R	69R	57L	35L	163R	235R	76L	72L	75L	295L	16R
10.	Myristilated membrane protein	53R	55R	51L	88L	59L	67L	158R	7L	8L	8L	118L, 458R	6R
11.	DNA pol Family B exonuclease	60R	63R	44L	128R	87R	135R	203L	19R	20R	22R	37L	120L
12.	DNA-dep RNA pol-II second largest subunit	62L	65L	43R	73L	46L	25L	25R	34R	33R	36R	428L	9R
13.	Ribonucleotide reductase small subunit	67L	71L	38R	47L	26L	27R	41L	24R	26R	27R	376L	48L
14.	Ribonuclease III	80L	85L	25R	84L	55L	137R	187R	87R	83R	85R	142R	101R
15.	Proliferating cell nuclear antigen	84R	90R	20L	68L	41L	3L	197L	112R	**103R** ^b^	109R	436L	60L
16.	Major capsid protein	90R	96R	14L	72R	45R	147L	43L	6L	7L	7L	274R	14L
17.	Putative XPPG-RAD2-type nuclease	95R	101R	10L	97L	66L	191R	169R	27L	28L	30L	369L	76L
18.	Serine-threonine protein kinase	19R	19R	80L	39L	21L	10L	45R	55L	53L	56L	380R	10L
19.	Serine-threonine protein kinase	57R	59R	47L	150L	100L	143L	178L	13R	13R	15R	98R	98L

The *Iridoviridae* core genes (Eaton et al., 2007). ^a^ ORFs that have been added or altered are highlighted in bold, ^b^ Potentially frameshifted ORF.

**Table 2:** Additional *Iridoviridae* core genes identified after genome re-analysis.

Newly Characterized Gene Name ^a^	FV3	TFV	ATV	SGIV	GIV	LCDV-1	LCDV-C	ISKNV	RBIV	OSGIV	IIV-6	MIV
Myristilated membrane protein	2L	**2L** ^b^	1L	19R	4R	160L	38R	**90.5L**	85L	**88.5L**	337L	47R
Unknown	12L	12L	87R	118R	80R	108L	100L	96L	**89.5L** ^b^	93L	287R	56L
Transcription elongation factor TFIIS	81R	86R	24L	85R	56R	171R	115R	29L	**29.5L** ^b^	32L	349L	55R
Deoxynucleoside kinase	85R	**91.5R**	19L	67L	40L	136R	027R	32R	31R	34R	143R	29R
Erv1/Alr family	88R	94R	16L	70R	43R	106L	142L	43L	**43.5L**	45L	347L	96R
Immediate early protein ICP-46	91R	97R	13L	162L	108L	47L	162R	115R	**108.5R**	112R	393L	39R
Hypothetical protein-Clostridium tetani	94L	100R	11L	98R	67R	19R	153L	86R	**82.5R**	**84.5L**	307L	33L

The *Iridoviridae* core genes identified subsequent to genome re-analysis (Eaton et al., 2007). ^a^ ORFs that have been added or altered are highlighted in bold. ^b^ Potentially frameshifted ORF.
